# Cell-Free DNA as Biomarker in Oral Squamous Cell Carcinoma: Dynamics, Mutational Landscape and Clinical Implications

**DOI:** 10.3390/cells15060568

**Published:** 2026-03-23

**Authors:** Pedro Veiga, Leonor Barroso, Luís Miguel Pires, Carolina Mano, Francisco Caramelo, Isabel Marques Carreira, Ilda Patrícia Ribeiro, Joana Barbosa de Melo

**Affiliations:** 1 Cytogenetics and Genomics Laboratory, Institute of Cellular and Molecular Biology, Faculty of Medicine, University of Coimbra, 3004-531 Coimbra, Portugal; pveiga@fmed.uc.pt (P.V.); lmlmpires@gmail.com (L.M.P.); carolinasequeiramano@gmail.com (C.M.); icarreira@fmed.uc.pt (I.M.C.); mmelo@fmed.uc.pt (J.B.d.M.); 2 Maxillofacial Surgery Department, Unidade Local de Saúde de Coimbra, 3004-561 Coimbra, Portugal; leonorbarroso@gmail.com; 3 Laboratory of Biostatistics and Medical Informatics, Coimbra Institute for Clinical and Biomedical Research (iCBR), Faculty of Medicine, University of Coimbra, 3004-531 Coimbra, Portugal; fcaramelo@fmed.uc.pt; 4 Center of Investigation on Environment Genetics and Oncobiology (CIMAGO), Coimbra Institute for Clinical and Biomedical Research (iCBR), Faculty of Medicine, University of Coimbra, 3004-531 Coimbra, Portugal; 5 Center for Innovative Biomedicine and Biotechnology (CIBB), University of Coimbra, 3004-531 Coimbra, Portugal; 6 Clinical Academic Center of Coimbra (CACC), Hospitais da Universidade de Coimbra, 3004-561 Coimbra, Portugal

**Keywords:** oral squamous cell carcinoma, liquid biopsy, next-generation sequencing, cell-free DNA, biomarkers

## Abstract

Oral squamous cell carcinoma (OSCC) is a prevalent form of head and neck cancer that typically develops on the lip or within the oral cavity. Although there have been advances in early detection and treatment, the prognosis for patients, particularly those with advanced-stage disease, remains poor. Liquid biopsy, particularly through the analysis of cell-free DNA (cfDNA) in plasma and urine, has emerged as a promising tool for non-invasive cancer detection and monitoring. This study assessed cfDNA concentration dynamics in plasma and urine samples from 32 OSCC patients, with 5 undergoing genomic characterization by targeted next-generation sequencing (NGS). CfDNA levels were higher in patients compared to healthy controls and showed transient increases following treatment initiation, likely reflecting tumor cell death, followed by a gradual return to baseline. However, cfDNA concentrations were not significantly associated with tumor stage, recurrence, or progression-free survival. Targeted NGS analysis revealed a heterogeneous mutational landscape, identifying 76 variants across tumor tissue and initial cfDNA, with 30.3% shared between both sources. Recurrent hotspot mutations were detected in several important genes, including *TP53*, *PIK3CA*, *KRAS*, *APC*, and *FBXW7*. Urine cfDNA also captured several mutations absent from plasma or tissue, supporting its complementary value. These findings demonstrate that cfDNA analysis can dynamically reflect treatment response and capture tumor heterogeneity in OSCC. While informative, cfDNA quantification alone offers limited prognostic reliability, reinforcing the need for a multidimensional approach that includes genomic and clinical evaluation. Overall, this study supports the potential of liquid biopsy as a real-time, non-invasive tool for molecular monitoring and personalized management of OSCC patients.

## 1. Introduction

Oral squamous cell carcinoma (OSCC) accounts for approximately 90% of all oral malignancies and can arise in various parts of the oral cavity, including the lips, tongue, gums, and inner cheeks [1,2]. It is the most prevalent form of head and neck cancer (HNC) and can be easy to miss in its early stages. Therefore, it remains a global health problem associated with a high mortality rate, especially in combination with risk factors such as smoking, heavy drinking and HPV infections [2].

Oral cancer often arises from a complex interaction of genetic mutations and epigenetic changes that disrupt key cellular pathways. Mutations in genes such as *TP53*, *EGFR*, *CDKN2A* and *PIK3CA* that alter key signaling pathways, including PI3K/AKT/mTOR, TP53 and WNT/β-catenin, drive tumor growth and resistance to therapy [3].

Liquid biopsy is a powerful tool for improving the detection and management of oral cancer. Unlike traditional tissue biopsies, which are invasive and may not capture the full genetic diversity of a tumor, liquid biopsy analyzes molecular components originating in the tumor that may be present in the saliva, blood and urine of cancer patients. When combined with sequencing technologies, liquid biopsies can identify a wide range of genetic mutations and molecular alterations enabling a more comprehensive tumor characterization [3,4]. The analysis of cell-free DNA (cfDNA) levels alongside the mutational profile obtained through next-generation sequencing (NGS) is proving to be a promising approach because elevated cfDNA concentrations often reflect tumor burden and disease progression, while sequencing can reveal specific genetic alterations [5]. These two approaches allows clinicians to monitor tumor dynamics in real time and identify emerging mutations that confer resistance to treatment without the need of repeated tissue biopsies [4].

Although several studies have explored the utility of cfDNA in various cancers, data on cfDNA dynamics and mutation analysis in oral cancer, particularly through blood and urine liquid biopsy, remain scarce [6,7,8]. Moreover, few studies combine longitudinal cfDNA analysis with mutational profiling across multiple treatment timepoints [9,10].

In this study, we aimed to evaluate the concentration and dynamics of cfDNA in plasma and urine samples from patients with oral squamous cell carcinoma. We further investigated cfDNA kinetics across multiple treatment timepoints to assess their potential utility as biomarkers for therapeutic response, recurrence, and disease progression. Additionally, we performed targeted next-generation sequencing (NGS) to characterize the mutational landscape of cfDNA and tumor tissue, aiming to identify clinically relevant mutations and explore their evolution in both plasma and urine. Through this integrative approach, we seek to determine the feasibility and clinical relevance of liquid biopsy for disease monitoring in OSCC.

## 2. Materials and Methods

### 2.1. Study Population

The study was approved by the Committee on Ethics in Research of the Faculty of Medicine of the University of Coimbra (CE-128/2018). All procedures were conducted according to the Declaration of Helsinki, and patients provided written informed consent. We analyzed 32 OSCC patients, and 5 were selected for further, more detailed genomic characterization, based on the availability of sufficient high-quality cfDNA across multiple timepoints and the diversity of clinical presentations represented within the cohort. They were recruited between June 2019 and January 2021 from the Maxillofacial Surgery Unit of the Coimbra Hospital and University Center, CHUC, EPE, Portugal. Diagnosis and staging were performed in accordance with the 8th edition of the American Joint Committee on Cancer’s TNM (tumor-node-metastasis) staging system. The detailed characterization of the study cohort is presented in Table 1. The follow-up periods ranged from 34 to 64 months. These patients provided tumoral tissue, blood and urine samples collected at several timepoints during their clinical course (Supplementary Material—Table S1). Tumor tissue samples were preserved in RNAlater and stored at −20 °C until use. Approximately 10 mL of blood was collected in cfDNA blood collection tubes (Streck, Nebraska). Blood was centrifuged at 1600× *g* for 10 min at room temperature (RT). The obtained plasma was centrifuged again at 3200× *g* for 20 min, and the cell-free supernatant was stored at −80 °C. Approximately 50 mL of urine was collected into an empty sterile nuclease-free urine collection cup. Urine was transferred to a sterile nuclease-free 50 mL tube and centrifuged at 500× *g* for 10 min at RT. The upper phase was then transferred to a fresh sterile nuclease-free 50 mL tube and centrifuged at 2000× *g* for 10 min at RT. Next, approximately 42 mL of urine were transferred to 2 fresh, sterile, nuclease-free 50 mL tubes (21 mL of urine per tube) and these aliquots were stored at −80 °C.

Controls, consisting of blood samples from five patients without a cancer diagnosis, were followed in the outpatient unit of the maxillofacial department. Regarding the characteristics of the five control subjects, three were male and two were female, with a mean age of 58.2 years (range 55–64 years). Only one of these controls was a current smoker, with the remaining presenting a previous history of smoking, except for one. Moreover, only one of these subjects had habits of alcohol consumption.

### 2.2. DNA Extraction

Isolation of cfDNA from plasma and urine samples was performed using the QIAamp Circulating Nucleic Acid Kit (Qiagen, Germany) according to the manufacturer’s instructions. Total cfDNA quantification was performed using Invitrogen Qubit dsDNA HS Assay Kit and Qubit 3.0 Fluorometer (Life Technologies, Carlsbad, CA, USA) according to manufacturer’s instructions. Genomic DNA from fresh-frozen patient tissues was extracted using a High Pure PCR Template Preparation Kit (Roche GmbH, Mannheim, Germany) according to the manufacturer’s instructions. The DNAs were quantified using a Nanodrop 1000 Spectrophotometer (Thermo Scientific, Waltham, MA, USA).

### 2.3. Next-Generation Sequencing (NGS)

NGS was performed using tumor tissue DNA and cfDNA extracted from plasma samples collected at different timepoints during the monitoring period from five oral cancer patients (Supplementary Material—Table S2). For preparation of the NGS library, a specific panel to detect ctDNA from colorectal and other related gastro-intestinal cancers—Oncomine Colon cfDNA assay (Thermo Fisher Scientific, Waltham, MA, USA)—was used, since an OSCC-specific panel was not available. Overall, this assay allows for the analysis of SNVs and short indels that are frequently mutated in colorectal and gastrointestinal cancers, covering a total of fourteen genes (*AKT1*, *BRAF*, *CTNNB1*, *EGFR*, *ERBB2*, *FBXW7*, *GNAS*, *KRAS*, *MAP2K1*, *NRAS*, *PIK3CA*, *SMAD4*, *TP53* and *APC*) with more than 240 hotspots. All the library preparation procedures were performed according to the manufacturer’s instructions. Each library was barcoded with adapters, enabling the multiplexed sequencing of multiple samples on the same chip. Barcoded libraries were quantified using Invitrogen Qubit dsDNA HS Assay Kit and Qubit 3.0 Fluorometer (Life Technologies, Carlsbad, CA, USA) and pooled to a normalized concentration of 50 pM. Next, template preparation and chip loading into an Ion 530 Chip were performed using the Ion 520 & Ion 530 Kit and Ion Chef System (Thermo Fisher Scientific, Waltham, MA, USA), according to the manufacturer’s instructions. Finally, the sequencing run was planned on the Torrent Suite Software v5.10.1, and the prepared chip was loaded into the Ion S5 System (Thermo Fisher Scientific, Waltham, MA, USA) for sequencing. Data analysis was performed using the Ion Reporter Software v5.14 (Thermo Fisher Scientific, Waltham, MA, USA).

### 2.4. Data Analysis

Data analysis was carried out using R Statistical Software v4.5.1. The mean and standard deviation were used to quantify the cfDNA data. Samples presenting extremely high cfDNA concentrations, considered outliers and likely to bias summary statistics, were excluded from the analysis. Progression-Free Survival (PFS) in patients with high versus low cfDNA concentration was analyzed using the Kaplan-Meier method. Differences between groups were assessed using the log-rank test.

## 3. Results

### 3.1. Cell-Free DNA Dynamics

#### 3.1.1. cfDNA Concentration in Patients vs. Controls

**Figure 1:**
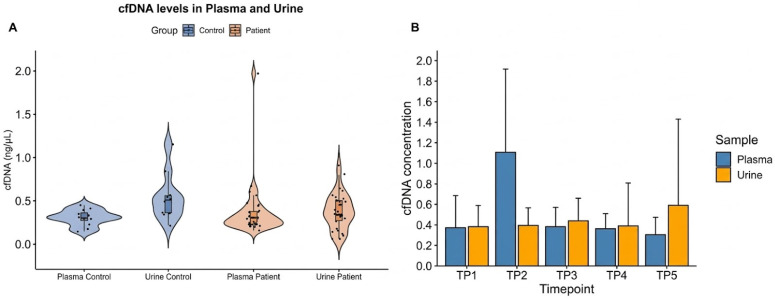
cfDNA concentration analysis of the 32 patients. (**A**) cfDNA concentration distribution between control (*n* = 5) and patients (*n* = 32) on plasma and urine samples. Violin plots display the distribution of cfDNA concentrations (ng/µL) across sample types: plasma and urine control vs. patient samples. Boxplots indicate the interquartile range with the median shown as a horizontal line. (**B**) cfDNA concentration dynamics across the different timepoints in OSCC patients (*n* = 32). Mean cfDNA concentration was calculated using data until the TP5, as this was the last timepoint common to all 32 OSCC patients. Bar plots represent the mean cfDNA concentration (ng/µL) measured at five timepoints (TP1–TP5). Blue bars correspond to plasma samples, and orange bars correspond to urine samples. Error bars indicate standard deviation. cfDNA levels fluctuate over time, with a notable increase in plasma cfDNA concentration at TP2 compared to other timepoints.

In the pre-treatment (TP1), average plasma cfDNA concentrations were slightly higher in patients (*n* = 32) than in healthy controls (*n* = 5) (Figure 1A). The mean cfDNA concentration in patient plasma samples was 0.372 ± 0.312 ng/µL (range: 0.157–1.97), whereas in the control group, it was 0.305 ± 0.088 ng/µL (range: 0.144–0.449). In urine samples, the average cfDNA concentration in patients was 0.382 ± 0.205 ng/µL (range: 0.058–0.806), while in controls, it was 0.540 ± 0.260 ng/µL (range: 0.213–1.15).

#### 3.1.2. cfDNA Concentration over Timepoints

Overall, cfDNA levels remained relatively stable across different timepoints, excepted for a marked increase following treatment (Figure 1B). The mean plasma cfDNA concentration rose from 0.372 ± 0.312 ng/µL at baseline (TP1) to 1.106 ± 0.811 ng/µL post-treatment initiation (TP2). Subsequently, cfDNA levels gradually decreased, approaching baseline values. In urine samples, cfDNA concentrations showed minimal fluctuation over time, with a slight increase observed between the TP4 and TP5 timepoints.

#### 3.1.3. cfDNA Concentration by Tumor Stage

**Figure 2:**
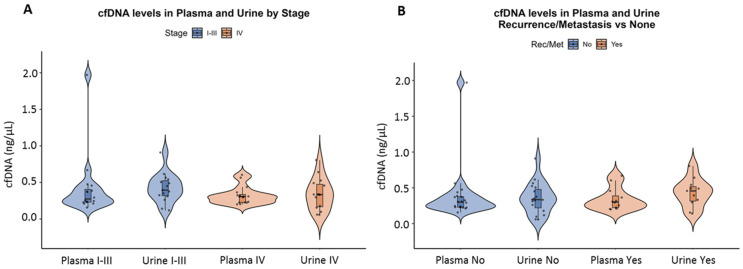
Comparison of circulating cell-free DNA (cfDNA) concentrations in plasma and urine between patients with stage I–III and stage IV disease at TP1 (**A**). cfDNA concentrations in plasma and urine in patients with or without recurrence/metastasis (Rec/Met) at TP1 (**B**). Data is presented as violin plots showing the distribution, median, and interquartile range for each group.

When stratified by tumor stage, mean cfDNA levels were slightly higher in early-stage patients (Stage I–III) than in advanced-stage patients (Stage IV), both in plasma and in urine (Figure 2A). In early-stage patients, the mean cfDNA concentration was 0.423 ± 0.419 ng/µL (range: 0.157–1.97) in plasma and 0.397 ± 0.191 ng/µL (range: 0.118–0.91) in urine. In contrast, advanced-stage patients exhibited lower cfDNA levels: 0.322 ± 0.117 ng/µL (range: 0.2–0.604) in plasma and 0.341 ± 0.209 ng/µL (range: 0.058–0.806) in urine.

#### 3.1.4. cfDNA Concentration in Patients with Metastasis or Recurrence

Comparison of cfDNA levels between patients who developed metastasis or recurrence and those who did not revealed no substantial differences in mean cfDNA concentrations (Figure 2B).

#### 3.1.5. cfDNA Concentration and Patient Survival

We performed a Kaplan-Meier survival analysis to compare progression-free survival (PFS) between patients with high and low cfDNA levels. The PFS probability curves for the high cfDNA and low cfDNA groups were largely overlapping over the follow-up period (Supplementary Material—Figure S1). The median PFS was not significantly different between the two groups. A log-rank test was performed to assess differences in survival distributions, with a *p*-value of 0.8, indicating no statistically significant difference in PFS between patients with high versus low cfDNA levels in this cohort.

#### 3.1.6. cfDNA Dynamics over Treatment Timepoints in the Five Patients Analyzed by NGS

Cell-free DNA concentrations were assessed in the five patients analyzed by NGS (ID: P1, P2, P3, P4, and P5) across multiple timepoints before and after treatment initiation. All patients subsequently underwent next-generation sequencing (NGS) analysis of tissue and cfDNA samples at multiple timepoints.

**Figure 3:**
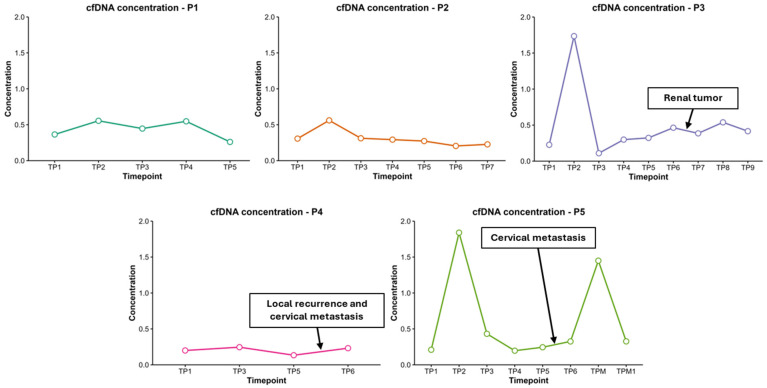
cfDNA concentration across different timepoints in the five patients analyzed by NGS (P1–P5). The *x*-axis represents the sampling timepoints and the *y*-axis indicates the cfDNA concentration. Arrows indicate timepoints corresponding to clinically documented disease events: renal tumor detection in patient P3 (between TP6 and TP7), local recurrence with cervical metastasis in patient P4 (between TP4 and TP5), and cervical metastasis in patient P5 (between TP5 and TP6), illustrating the temporal relationship between cfDNA dynamics and clinically documented disease progression.

In all five cases, an increase in cfDNA concentration was observed immediately following treatment initiation compared to baseline levels (Figure 3). The extent and duration of this elevation varied between patients.

Patients P1 and P2 demonstrated moderate increases in cfDNA levels immediately after treatment initiation (from 0.365 to 0.555 ng/µL and from 0.307 to 0.561 ng/µL, respectively), followed by a gradual decline at subsequent timepoints. These patterns may reflect an initial treatment response with subsequent stabilization. Notably, patient P2 did not develop metastasis or recurrence and remains the only patient alive, which aligns with the relatively stable cfDNA levels observed after surgery and throughout follow-up.

Patient P3 and patient P5 exhibited the most pronounced increases in cfDNA levels post-treatment, with concentrations rising from 0.227 ng/µL to 1.735 ng/µL and from 0.210 ng/µL to 1.840 ng/µL, respectively. Notably, P5 also showed a secondary elevation in cfDNA concentration at a later timepoint (1.450 ng/µL), suggesting either continuous tumor DNA shedding or potential disease progression.

In contrast, patient P4 maintained consistently low cfDNA levels throughout follow-up, with minimal variation from baseline (0.200 ng/µL pre-treatment to 0.246 ng/µL post-treatment). However, toward the end of the follow-up period, a slight increase in cfDNA levels was observed. This rise between TP5 and TP6 coincides with the diagnosis of a local recurrence and cervical metastasis.

In patients P3, P4, and P5, subtle alterations in cfDNA profiles were detected preceding the clinical confirmation of recurrence or metastasis. These findings suggest that longitudinal cfDNA detection may provide an effective approach for early detection of disease progression and improve clinical management.

### 3.2. Liquid Biopsy

#### 3.2.1. Mutational Landscape

We analyzed somatic mutations identified in tumor tissue and liquid biopsy samples from five patients. For each patient, tissue samples and multiple timepoints of plasma and urine with sufficient cfDNA were sequenced for analysis (Supplementary Material—Figure S2).

**Figure 4:**
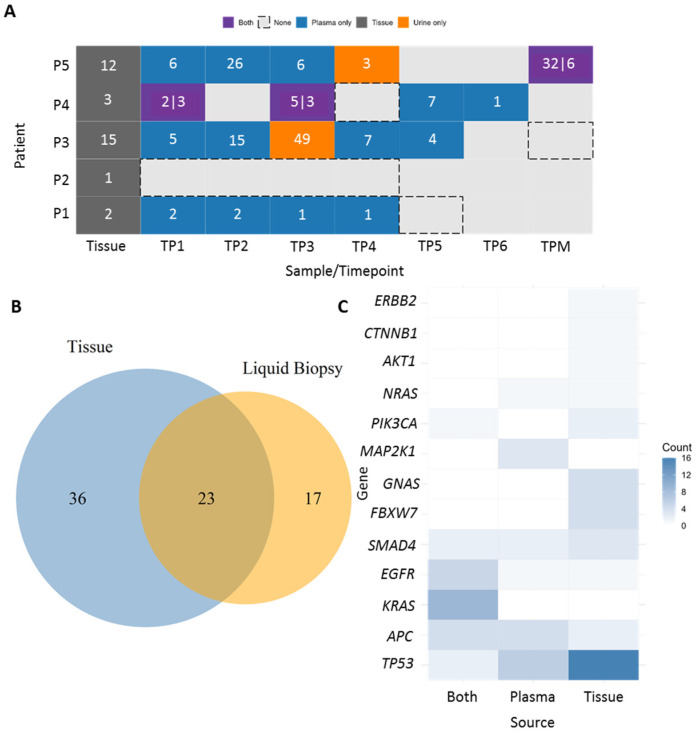
Mutational landscape overview. (**A**) Heatmap showing the number of hotspot mutations per patient and timepoint. Colored rectangles indicate the sequenced sample type: plasma (blue), urine (orange), both plasma and urine (purple), and tissue (dark gray). Numbers within each rectangle show the number of hotspot mutations. For samples with mutations in both plasma and urine, the first number corresponds to plasma and the second to urine. Black rectangles indicate sequenced samples with no hotspot mutations detected. (**B**) Venn diagram showing the total number of mutations (both novel and hotspot) detected by NGS in tumor tissue and in plasma-liquid biopsy. (**C**) Heatmap showing the number of mutations detected in each gene according to sample type. The color scale indicates the number of mutations detected per gene, with white representing 0 mutations, light blue representing 1–3 mutations, medium blue representing 4–8 mutations, and dark blue representing more than 8 mutations.

The mutational landscape of our cohort was highly heterogeneous, characterized by single-nucleotide variants (SNVs), primarily missense mutations, along with a smaller proportion of nonsense mutations. We detected various novel mutations, predicted as oncogenic, likely benign, and variants of uncertain significance (VUS). In addition, several recurrent hotspot mutations were identified in key cancer-associated genes, which will serve as the main focus of our subsequent analyses (Figure 4A and Table S3).

A total of 76 mutations (novel and hotspot) were detected across all pre-treatment samples (tissue and plasma-TP1). Of these, 36 mutations (47.4%) were detected exclusively in tumor tissue, 17 mutations (22.4%) exclusively in plasma-liquid biopsy, and 23 mutations (30.3%) were identified in both tissue and liquid biopsy (Figure 4B). The most frequently mutated gene was *TP53* with 24 mutations, 16 of which were found exclusively in tumor tissue, 6 in liquid biopsy, and 2 shared between both sources. Other commonly mutated genes included *KRAS* (9 mutations, all shared between tissue and liquid biopsy), *EGFR* (5 mutations; 1 in tissue, 1 in liquid biopsy, and 3 in both), and *APC* (4 mutations shared across all sources) (Figure 4C).

Some mutations were detected exclusively in tissue, including in the *FBXW7*, *CTNNB1*, and *GNAS* genes, whereas *MAP2K1* mutations were observed only in liquid biopsy. Several genes, including *AKT1*, *ERBB2*, *NRAS*, and *PIK3CA*, showed mutations but did not follow a consistent pattern.

#### 3.2.2. Dynamic Mutational Landscapes from Patients P1-P5

**Figure 5:**
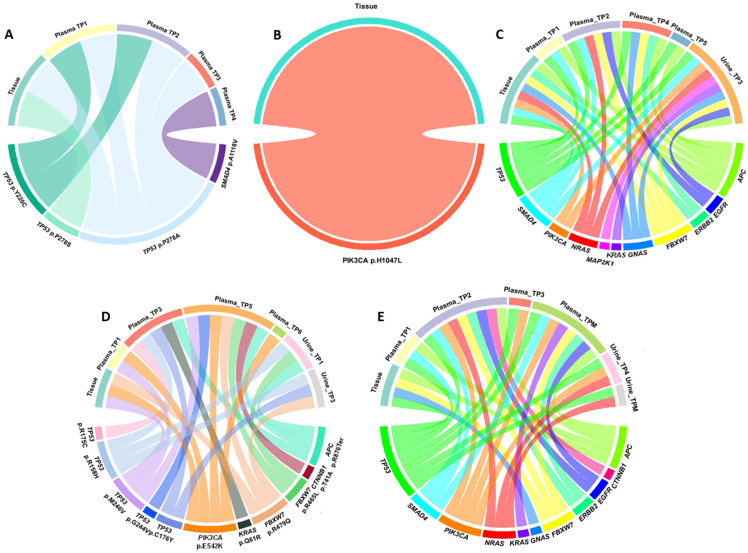
Chord plots illustrating the distribution of the mutations detected in P1–P5 across the different sample types and timepoints. Each link represents the presence of a specific mutation in both tissue and plasma cfDNA. (**A**) Mutations in TP53 (p.Y220C and p.P278A) were consistently detected across multiple plasma timepoints, indicating persistent tumor alterations. The SMAD4 p.A118V mutation was identified only in plasma at later timepoints, suggesting the emergence of a subclonal variant during disease progression. (**B**) The only hotspot mutation identified in the tissue sample was PIK3CA p.H1047L. No hotspot mutations were detected in any of the plasma samples. (**C**,**E**) Due to the high number of mutations observed in these cases, each colored link (chord) represents the presence of one or more mutations within the corresponding gene in each sample, rather than individual mutations. (**D**) Several TP53 gene mutations were observed in P5 across multiple sources and timepoints.

Targeted sequencing of tumor tissue and serial plasma samples from patient 1 (P1) revealed dynamic clonal evolution in *TP53* and the emergence of additional driver mutations (Figure 5A). At baseline, tumor tissue showed a dominant *TP53* mutation, p.P278S, with a high variant allele frequency (VAF, 38.8%), accompanied by a minor *TP53* mutation, p.P278A.

Analysis of plasma cfDNA demonstrated marked temporal changes in the mutational landscape. At TP1, *TP53* p.P278A persisted at low frequency, while *TP53* p.Y220C emerged, reflecting an early subclone detected in plasma and not observed in tissue. By TP2, *TP53* p.P278A exhibited a rapid increase in frequency, whereas p.Y220C declined, indicating selective expansion of the P278A clone in circulation. At TP3, *TP53* p.P278A became the dominant circulating mutation, consistent with clonal expansion over time.

Additionally, a *SMAD4* mutation, p.A118V, was detected at TP4, representing a late-arising clone detectable only in plasma, suggesting the emergence of a subclonal variant during disease progression. We analyzed somatic mutations identified in tumor tissue and liquid biopsy samples from five patients. For each patient, tissue samples and multiple timepoints of plasma and urine with sufficient cfDNA were sequenced for analysis (Supplementary Material—Figure S2).

In patient P2, at baseline 12, novel mutations were detected in tumor tissue, including 1 hotspot oncogenic mutation, *PIK3CA* p.H1047L, identified exclusively in the tumor sample (Figure 5B). In plasma (TP1), 11 novel mutations were detected, eight of which were shared between the two sources. In contrast, plasma samples uniquely harbored four novel *MAP2K1* mutations. At later timepoints, a novel likely oncogenic TP53 mutation and a *SMAD4* mutation were detected in plasma, both of which were absent in subsequent collections.

Targeted sequencing of tumor tissue, plasma, and urine samples from patient 3 (P3) revealed a complex mutational landscape with alterations in several genes, including *TP53*, *APC*, *FBXW7*, *SMAD4*, *ERBB2*, *EGFR*, *NRAS*, *KRAS*, *PIK3CA*, *MAP2K1*, and *GNAS* (Figure 5C).

At baseline tumor tissue, multiple oncogenic mutations were identified, including truncating mutations in *APC* (p.Y935Ter), missense mutations in *TP53* (p.R273H; p.R249M; p.R248Q; p.G245S; p.V216M), *FBXW7* (p.R505C; R582L), *SMAD4* (p.A118V; p.R361H), *ERBB2* (p.S310F), and *GNAS* (p.R201C; p.R201H). Of note, *TP53* p.R248Q was detected at a higher VAF (24.9), consistent with a dominant clonal event.

In serial plasma samples, dynamic changes in the mutational profile were observed. At TP1, truncating *APC* (p.E1306Ter) and multiple *TP53* hotspot mutations were detected alongside *SMAD4* p.A118V. At TP2, additional driver mutations emerged, including *NRAS* p.Q61R, *FBXW7* mutations (p.R689W, p.R505C, and p.R465H), *APC* p.R876Ter, *EGFR* p.R451C, and multiple *TP53* mutations spanning codons 173–273. At TP3, recurrent *APC* truncating mutation (p.Y935Ter), *FBXW7* p.S582L, *TP53* missense mutations (p.R249S, p.G244V, p.V173M), and *ERBB2* p.R896C were observed. At TP5, plasma retained evidence of *APC* truncation (p.R1114Ter) and also *TP53* mutations (p.V272; p.G245C; p.R175H).

Analysis of urine cfDNA demonstrated broad concordance with tissue- and plasma-derived profiles, capturing mutations in *APC* (e.g., p.R805Ter; p.R1450Ter), *FBXW7*, *EGFR* (p.R451C, p.S492R), *KRAS* (p.Q61R; p.G12C), *NRAS* p.G12V/C, *PIK3CA* p.Q546K and *MAP2K1* p.K57N. A wide spectrum of *TP53* mutations were also identified, including p.R273H, p.R248W, p.R175L, and p.R175C, among others. Notably, several mutations (*TP53:* p.R175L, GNAS: p.R201C, *SMAD4*: p.G510V) were detected exclusively in urine, indicating a potential complementary role of urine liquid biopsy.

At baseline, tissue analysis of patient P4 revealed three hotspot mutations: *TP53* p.M246V, *PIK3CA* p.E542K and *FBXW7* p.R479Q.

Longitudinal analysis of plasma cfDNA revealed dynamic changes in the mutational landscape across multiple time points (Figure 5D). At TP1, both *PIK3CA* p.E542K and *TP53* p.M246V were detected, mirroring the baseline tissue profile and indicating early release into the circulation. By TP3, additional mutations were observed, including *KRAS* p.Q61R, *APC* p.R876Ter, and multiple *TP53* mutations: p.C176Y, p.R175C, and p.R158H. This pattern suggests emerging subclonal diversity or ongoing tumor evolution.

The most diverse mutational profile was observed at TP5, with eight hotspot mutations identified: *CTNNB1* p.T41A, *PIK3CA* p.E542K, *FBXW7* p.R479Q and p.R465H, *APC* p.R876Ter, and three *TP53* mutations, including the original p.M246V and a new mutation p.G244V. At TP6, only *PIK3CA* p.E542K was detected.

Urine cfDNA analysis revealed additional insights. At TP1, mutations in *TP53* (p.R158H), *FBXW7* (p.R465H), and *APC* (p.R876Ter) were identified. By TP3, the presence of TP53 mutations (p.R158H and p.C176Y) had diminished, and *FBXW7* p.R479Q was detected at a low level.

Across all sample types and timepoints, several mutations persisted. *PIK3CA* p.E542K and *TP53* p.M246V were consistently detected in both tissue and plasma. In contrast, other *TP53* mutations appeared later in both plasma and urine, indicative of subclonal evolution. *FBXW7* mutations were detected in all sources, but at consistently low VAFs. The *APC* p.R876Ter mutation was recurrently detected in both plasma and urine.

In patient P5, at baseline, multiple pathogenic mutations were identified including *FBXW7* p.R465H, *APC* p.R1450Ter, *GNAS* p.R201S and p.R201C, and *TP53* mutations spanning codons 141–282, indicating early intratumoral heterogeneity.

In serial plasma samples, dynamic changes in mutational profiles were observed (Figure 5E). At TP1, *APC* truncating mutations (p.R805Ter; p.R876Ter) and *TP53* p.R280I were detected alongside *SMAD4* mutations (p.G386D; p.G510V) and *TP53* p.V173L. At TP2, additional driver mutations emerged, including *NRAS* p.G13C and p.G12V, *PIK3CA* p.Q546K and p.M1043I, *FBXW7* p.S582L, *APC* p.Y935Ter and p.E1353Ter, *EGFR* p.G465E, *ERBB2* p.S310F and p.R896C, and a broad spectrum of *TP53* mutations spanning codons 158–282. At TP3, recurrent *PIK3CA* mutations (p.M1043V/I), *TP53* p.R282W, p.G266V, p.R249S, and p.R158H and *ERBB2* p.R896C were observed.

Post-metastasis surgery plasma (TPM) retained low-frequency residual clones, including *APC* (p.R805Ter; p.R876Te; p.Y935Ter; p.E1353Ter; p.E1577Ter), *TP53* (multiple hotspot mutations), *FBXW7* (p.W526R; p.R505C; p.R465H), *PIK3CA* (p.Q546K; p.M1043I), and other mutations in *ERBB2*, *SMAD4*, *EGFR*, and *KRAS* genes.

Analysis of urine cfDNA (TP4) demonstrated concordance with plasma and tissue profiles, capturing high-frequency mutations including NRAS p.G13C, *PIK3CA* p.E545K, and *TP53* p.C242F. Additional *TP53* mutations (p.G266R, p.G245C, p.H214R, p.I195T, p.P152L) in the post-metastasis surgery urine sample were also detected.

## 4. Discussion

Concentration and cell-free DNA (cfDNA) dynamics in plasma and urine samples from oral cancer patients were evaluated compared with healthy controls, with additional stratification by tumor stage, treatment timeline and clinical outcomes.

At baseline (TP1), patients demonstrated a slightly higher plasma cfDNA concentration than healthy controls, consistent with previous studies linking elevated cfDNA levels to the presence of cancer cells shedding DNA into the bloodstream [11]. Regarding oral cancer, two studies also report higher levels of cfDNA in OSCC patients when compared to controls [6,7].

Urine cfDNA levels were also elevated in our cohort, reflecting differences in cfDNA clearance and physiological variability. Although it has been reported that cancer patients with urologic and non-urological cancers may have higher cfDNA concentrations, there are not many studies evaluating these cfDNA levels in the urine of OSCC patients [12].

Using cfDNA quantification in predicting therapeutic response has been investigated in several studies and it is reported that the reduction in cfDNA in plasma is associated with a better and sustained response to treatment in various types of cancer [13,14]. In patients with locally advanced squamous cell head and neck cancer, higher levels of cfDNA after or before treatment indicated incomplete tumor eradication and poor locoregional progression-free survival [14]. However, it is important to note that while cfDNA quantification offers significant potential as a non-invasive biomarker for cancer diagnosis, prognosis and treatment responses it lacks clinical validation. The concentration of cfDNA in plasma can be influenced by various non-cancerous conditions, such as inflammation, cardiovascular disease, as well as following physical exercise [15,16]. In that sense, the genomic analysis of cancer-specific mutations in ctDNA, such as through NGS, seems to be crucial for a correct evaluation.

Regarding cfDNA levels across the different timepoints, a significant and consistent rise in plasma cfDNA was observed immediately after treatment initiation in most patients, followed by a gradual return to baseline. This transient elevation likely reflects tumor cell death induced by therapy, with a subsequent decrease suggesting treatment efficacy or tumor regression, likely due to tissue manipulation during surgery [17].

In contrast, urine cfDNA levels showed less pronounced temporal changes, possibly due to differences in the kinetics of cfDNA elimination via the urinary tract, lower stability, or differences in shedding from the tumor site [18,19].

These dynamic cfDNA profiles support its use as a real-time biomarker for assessing treatment response, potentially contributing to more personalized treatments.

No significant differences in baseline mean cfDNA concentrations were observed between patients who developed metastasis or recurrence and those who did not. Although some studies show that higher cfDNA levels are often associated with disease recurrence or metastasis across various cancer types [20,21]. Our findings suggest that cfDNA concentration alone may not serve as a reliable standalone biomarker for metastasis or recurrence detection, at least within this small cohort of OSCC patients. The lack of significant differences could be attributed to several factors, including limited sample size, heterogeneity in tumor biology across patients, and potential confounding by other clinical or molecular factors.

We observed higher mean plasma and urine cfDNA concentrations in early-stage disease than in later stages, which contrast with the existing literature, which reports higher cfDNA levels in late-stage patients [22]. This unexpected pattern could be explained by several factors. Total cfDNA reflects DNA from multiple sources and not only tumor shedding, but also health conditions related to some patients may contribute to elevate the cfDNA levels. In addition, inter-individual differences in cfDNA clearance and in tumor physiology may alter cfDNA shedding [23]. Thus, discrepancies can occur, even among patients with tumors of the same type and stage, even though it is expected that late-stage patients have higher levels of cfDNA [24]. Our results are in line with those of Shukla et al. (2013), who also concluded that cfDNA levels were not related to stage [25]. Previous studies in head and neck cancer had the same results [26]. Altogether, although these findings seem to reveal that the concentration of cfDNA might not be as useful in distinguishing between early- and late-stage patients in oral cancer as it is for other cancer types, the fact that patients in early stages of the disease revealed cfDNA levels similar to late-stage patients seems to be encouraging for the early detection of oral cancer through liquid biopsies.

We also investigated whether baseline cfDNA levels were associated with progression-free survival outcomes in patients. Contrary to our initial hypothesis, the Kaplan-Meier analysis revealed no significant difference in PFS between patients with high and low cfDNA levels (*p* = 0.8). These findings suggest that cfDNA levels, as stratified in this analysis, may not serve as a predictive biomarker for PFS in this cohort.

Several factors could account for the lack of association observed. First, the sample size may have limited the statistical power to detect a difference. Additionally, disease heterogeneity among patients can interfere with the relationship between cfDNA levels and clinical outcomes. Furthermore, not all patients underwent the same therapeutic approach, introducing additional variability that may have influenced cfDNA dynamics and their correlation with clinical parameters.

Longitudinal tracking of cfDNA in the five patients analyzed by NGS revealed distinct profiles post-treatment, with some patients showing sharp transient increases and others exhibiting minimal variation. These differences may reflect tumor biology, treatment response, or intrinsic factors. Patient 5 displayed a secondary cfDNA peak, potentially indicating ongoing tumoral activity or early recurrence, giving emphasis to the importance of continuous monitoring. In this case, this patient underwent surgery after developing cervical metastasis between the TP5 and the TP6 timepoint which could explain the secondary cfDNA elevation. Although subtle, an overall increase or trend toward higher cfDNA levels was generally observed before the clinical diagnosis of recurrence or metastasis, which is in line with previous studies in multiple cancer types [21,27,28]. In patients without disease progression, such as patient 2, cfDNA levels remained relatively low and stable across all timepoints.

Overall, these findings accentuate inter-individual variability in cfDNA kinetics following treatment initiation. Transient spikes in cfDNA levels observed in some patients may reflect tumor cell death and active DNA release into circulation, providing clinically relevant information when interpreted in conjunction with NGS-derived mutational profiles.

This study also provides comprehensive mutational profiling of these five oral cancer patients by integrating both tumor tissue and liquid biopsy analyses, including serial plasma and urine samples. Our analysis revealed that 30.3% of mutations were shared between tissue and liquid biopsy, showing partial concordance between these two types of samples. Nearly half of the mutations (47.4%) were exclusive to tumor tissue, while 22.4% were detected only in liquid biopsy, emphasizing that while tissue biopsy remains essential for initial diagnosis, liquid biopsy can uncover additional tumoral mutations that may be missed due to tumor heterogeneity, including subclonal populations or metastatic lesions not captured by the tissue biopsy or sampling limitations. These missed mutations could have significant clinical implications, as they may represent key drivers of tumor progression or resistance to therapy. For example, mutations that are exclusive to tumor tissue may be critical for predicting therapeutic response, but their absence in liquid biopsy samples could lead to underestimation of the tumor’s mutational burden or an incomplete understanding of its molecular landscape.

Several recurrent hotspot mutations were identified, particularly in *TP53*, *PIK3CA*, *APC*, and *FBXW7*, suggesting common pathways in oral tumorigenesis and progression. Notably, several cell signaling pathways involving these genes are frequently activated and upregulated, contributing to OSCC progression [29]. Consistent with their known roles as cancer drivers, OncoKB classification confirmed that the majority of these hotspot variants were annotated as oncogenic or likely oncogenic. The level of clinical evidence for therapeutic actionability was assigned according to the OncoKB evidence framework, with several mutations, including *TP53* hotspots, *PIK3CA* E542K/E545K, *KRAS* G12C, and *NRAS* Q61R, carrying level 1–3 evidence for targeted therapeutic relevance in various cancer contexts.

Mutations in the *TP53* gene are common in oral cancers, present in 40–70% of cases, leading to a non-functional protein product. The majority of these occur between exons 5 and 8, particularly in the DNA-binding domain. Several mutations, such as R175, G245, R248, R249, R273, and R282, were observed in both P3 and P5, aligning with previous reports of their frequent occurrence in oral cancer [30]. Mutations such as R175H and R273H have been linked to poor surgical outcomes and higher rates of recurrence, which shows the clinical significance of these mutations in predicting prognosis and guiding treatment decisions [31]. The *TP53* Y220C mutation, found in P1 and P5, has been previously reported in oropharyngeal squamous cell carcinoma [32].

Similarly, *PIK3CA* E542K and E545K mutations, found in patients P4 and P5, have also been reported in head and neck cancer patients, further supporting the role of PIK3CA mutations in oral cancer development and progression [33,34]. Additionally, the PIK3CA M1043I mutation has been associated with oral cancer [34]. The *PIK3CA* H1047L mutation, detected in patient P2, has been previously identified in head and neck cancers, associated with poor prognosis and resistance to therapies such as cetuximab [35,36].

*SMAD4* mutations are also often observed in cancers with disrupted TGF-β signaling. The *SMAD4* A118V detected in P1, P3 and P5 is already described in head and neck cancer patients and is considered an oncogenic hotspot [37].

In patient P3, no mutations were detected in the pre-treatment urine sample. However, subsequent timepoints revealed an increased number of mutations, including mutations shared with the primary tumor tissue and additional unique alterations. The later diagnosis of a renal tumor in this patient may explain the elevated mutational burden observed in the second urine sample, suggesting that liquid biopsy can also capture mutations related to concurrent malignancies, thereby improving our understanding of the mutational landscape in complex cases.

One of the main limitations of this study is the relatively small sample size, which may limit the generalizability of our findings. In addition, the sensitivity and specificity of cfDNA detection can be heavily influenced by the technology used and the quality of the plasma and urine samples collected. Variations in how samples are processed, extracted, and sequenced can introduce inconsistencies, potentially affecting the results. Also, regarding the urine results, a potential limitation of this study is that urine physicochemical properties can influence cfDNA recovery. As only the supernatant fraction was analyzed, potential variability in cfDNA recovery cannot be excluded. This kind of technical variability is a challenge, and without standardized protocols, it is difficult to ensure reproducibility and consistency of findings. Going forward, larger sample sizes and more uniform methodologies will be essential to improve the reliability and accuracy of liquid biopsies for oral squamous cell carcinoma.

This study demonstrates that cfDNA analysis in plasma and urine offers promising potential as a non-invasive biomarker for oral squamous cell carcinoma. Elevated cfDNA levels in patients compared with healthy controls, and dynamic changes following treatment, suggest its usefulness for monitoring therapeutic response. However, baseline cfDNA concentrations did not correlate with disease stage, progression-free survival, or recurrence, indicating that cfDNA quantification alone may not be a reliable prognostic marker.

Mutational analysis revealed partial concordance between tumor tissue and liquid biopsy samples, with the latter identifying additional mutations potentially missed in tissue analysis, thereby reinforcing its complementary role in capturing tumor heterogeneity. In this cohort of five patients, recurrently detected mutations in *TP53*, *PIK3CA*, *SMAD4*, and *FBXW7* are consistent with the established roles of these genes in oral tumorigenesis and may serve as a starting point for future investigations into their prognostic and therapeutic significance in larger OSCC cohorts.

Overall, this study supports the clinical relevance of cfDNA analysis as a dynamic and minimally invasive tool for monitoring disease progression and treatment response in OSCC. However, larger cohorts, standardized methodologies, and integration of cfDNA quantification with genomic and clinical data are required to validate its prognostic value and translate these findings into routine clinical practice.

## Figures and Tables

**Table 1:** Clinicopathologic characteristics of the 32 patients.

	Patients = 32
	N (%)
**Gender**	
Female	6 (18.75%)
Male	26 (81.25%)
**Age**	
30–50	6 (18.75%)
51–70	14 (43.75%)
>70	12 (37.50%)
**Tumor location**	
Tongue	7 (21.88%)
Retromolar trigone	3 (9.38%)
Buccal mucosa	7(21.88%)
Floor of the mouth	5 (15.63%)
Alveolar ridge	5 (15.63%)
Floor of the mouth/Alveolar ridge	1 (3.13%)
Tongue/Floor of the mouth	2 (6.25%)
Retromolar trigone/Tongue	1 (3.13%)
Buccal mucosa/Retromolar trigone	1 (3.13%)
**Stage**	
I	3 (9.38%)
II	5 (15.63%)
III	8 (25%)
IV	16 (50%)
**Smoking habits**	
Yes	10 (31.25%)
No	22 (68.75%)
**Alcohol consumption**	
Yes	19 (59.38%)
No	13 (40.63%)
**Treatment ^1^**	
S	6 (18.75%)
SRT	6 (18.75%)
SQTRT	17 (53.13%)
QTRT	2 (6.25%)
QT-QTRT	1 (3.13%)
**Metastasis or recurrence**	
Yes	11 (34.38%)
No	21 (65.63%)
**Clinical outcome**	
Alive	24 (75%)
Death due to disease	5 (15.63%)
Death from other causes	3 (9.38%)

^1^ S—surgery; RT—radiotherapy; QT—chemotherapy.

## Data Availability

The datasets generated during the current study are available from the corresponding author upon request.

## Supplementary Materials

The following supporting information can be downloaded at https://www.mdpi.com/article/10.3390/cells15060568/s1. Table S1: Biofluids sample collection timepoints, detailing collection phases from pre-treatment through long-term follow-up; Tabel S2: Clinicopathologic characteristics of the five patients analysed by NGS; Table S3: List of hotspot variants detected across the different sources and timepoints. Figure S1: Kaplan-Meier curves comparing progression-free survival (PFS) between patients with high and low levels of circulating cell-free DNA (cfDNA). Patients were stratified into two groups based on the median cfDNA levels: High cfDNA (blue) and Low cfDNA (orange). Figure S2: NGS analysis of samples from the five patients across timepoints. Colored rectangles indicate the sequenced sample type: plasma (blue), urine (orange), both plasma and urine (purple), and tissue (gray). Blank spaces indicate unavailable samples.

## Abbreviations

The following abbreviations are used in this manuscript:

OSCC	Oral Squamous Cell Carcinoma
cfDNA	Cell-free DNA
HNC	Head and neck cancer
NGS	Next generation sequencing
TNM	Tumor-node-metastasis
S	Surgery
RT	Radiotherapy
QT	Chemotherapy
PFS	Progression-free survival
SNV	Single-nucleotide variant
VUS	Variant of uncertain significance
VAF	Variant Allele Frequency
