# Supplementary material for: Cell-Free DNA as Biomarker in Oral Squamous Cell Carcinoma: Dynamics, Mutational Landscape and Clinical Implications
Source: Cells. 2026 Mar 23;15(6):568. doi: 10.3390/cells15060568 (PMC13026038; doi:10.3390/cells15060568)
Supplement: Supplementary file 1 [file cells-15-00568-s001.zip › Supplementary material.pdf]

Supplementary material

Table S1: Biofluids sample collection timepoints, detailing collection phases from pre-treatment through long-term follow-up

Timepoint	Time of collection
TP1 (pre-treatment)	Blood and urine collection days before surgery.
TP2 (post-treatment)	First blood and urine sample collection shortly after surgery (usually within the first week).
TP3 (2nd post-treatment)	Follow-up, blood and urine collection between 1-2 months after surgery.
TP4 (3rd post-treatment)	Second follow-up, collection around 3-6 months after surgery.
TP5-TP9 (4th to 9th post-treatment)	Additional timepoints (late follow-up), collection ≥ 6 months after surgery, 1 year, 2 years and 3 years after surgery, depending on patient availability.
TPM (post-metastasis surgery)	Blood and urine collection after surgery in patients who develop recurrence/metastasis.

Table S2: Clinicopathologic characteristics of the five patients analysed by NGS.

Patient	Gender	Age	Tumor location	Stage	Smoking habits	Alcohol consumption	Treatment	Metastasis or recurrence	Clinical outcome
P1	M	62	Tongue	IV	Yes	No	SQT	Yes (hypopharynx)	Deceased
P2	M	57	Tongue / Floor of the mouth	IV	Yes	No	SQTRT	No	Alive
P3	M	50	Floor of the mouth	IV	Yes	Yes	SQTRT	No*	Deceased
P4	M	59	Alveolar ridge	IV	Yes	Yes	SQTRT	Yes (Local recurrence)	Deceased
P5	M	66	Floor of the mouth	III	No	No	SQTRT	Yes (cervical metastasis)	Deceased

Legend: S – surgery; RT – radiotherapy; QT – chemotherapy; *the patient developed a renal and a later pancreatic tumor that were unrelated to the initial tumor.

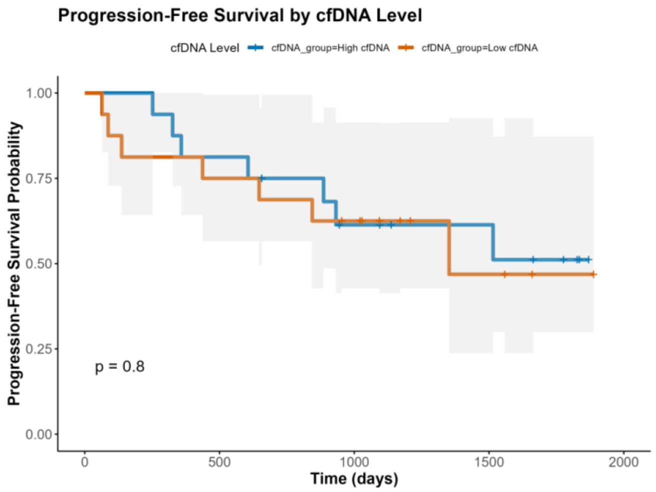

Figure S1: Kaplan-Meier curves comparing progression-free survival (PFS) between patients with high and low levels of circulating cell-free DNA (cfDNA). Patients were stratified into two groups based on the median cfDNA levels: High cfDNA (blue) and Low cfDNA (orange).

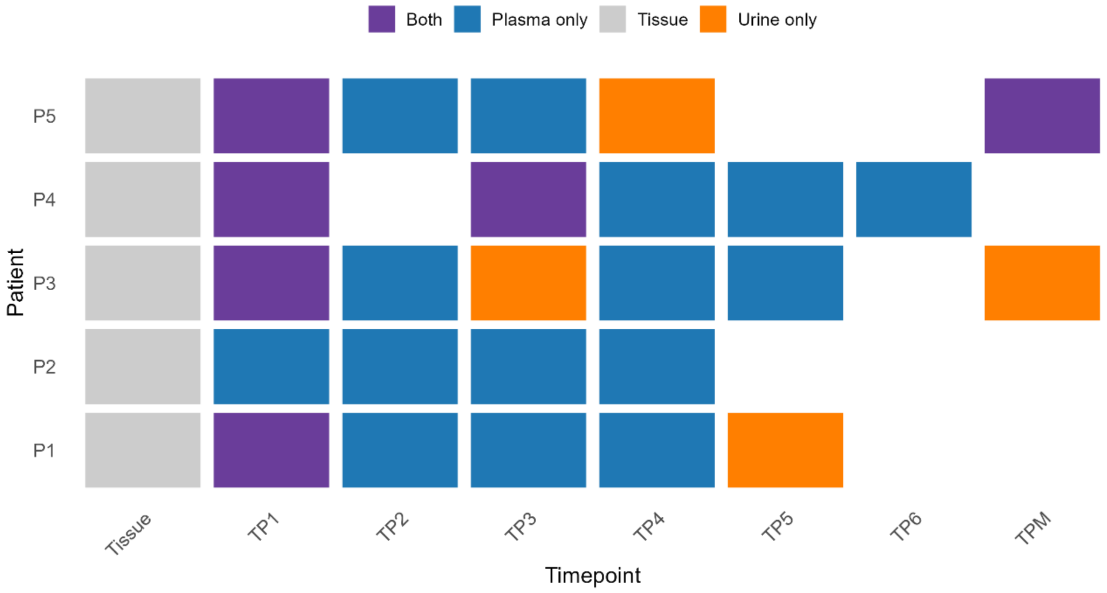

Figure S2: NGS analysis of samples from the five patients across timepoints. Coloured rectangles indicate the sequenced sample type: plasma (blue), urine (orange), both plasma and urine (purple), and tissue (grey). Blank spaces indicate unavailable samples.
